# Strategic discussion on funding and access to therapies targeting rare diseases in Spain: an expert consensus paper

**DOI:** 10.1186/s13023-023-02635-3

**Published:** 2023-02-24

**Authors:** N. Zozaya, J. Villaseca, F. Abdalla, A. Ancochea, I. Málaga, M. Trapero-Bertran, N. Martín-Sobrino, O. Delgado, P. Ferré, A. Hidalgo-Vega

**Affiliations:** 1 Health Economics Department, Weber, C/ Moreto 17, 5D, 28014 Madrid, Spain; 2 Federación Española de Enfermedades Raras (FEDER), Madrid, Spain; 3 Head of the Neuropediatrics Unit, Asturias Central University Hospital, Asturias, Spain; 4 Department of Basic Sciences, Universitat Internacional de Catalunya (UIC Barcelona), Barcelona, Spain; 5 Castilla y León Health Management Department, Technical Director of Pharmacy, Valladolid, Spain; 6 Pharmacy Service, Son Espases University Hospital, Palma, Balearic Spain; 7 Technical Advisor On Temporal Leave, Ministry of Health, Madrid, Spain; 8 President of Weber Foundation, Madrid, Spain

**Keywords:** Reimbursement, Funding, Access, Rare diseases, Orphan drugs, Spain, Reflection, Measures, Regulatory science

## Abstract

**Background:**

In recent years, significant advances have been made in the field of rare diseases (RDs). However, there is a large number of RDs without specific treatment and half of these treatments have public funding in Spain. The aim of the FINEERR project was to carry out a multidisciplinary strategic discussion on the challenge of funding and access to RD-targeted drugs in Spain, in order to agree on specific proposals for medium-term improvement and hence support decision-making in the Spanish National Healthcare System (SNHS).

**Results:**

The FINEERR Project was organized around a CORE Advisory Committee, which provided an overview, agreed on the design and scope of the project, and selected the members within each of four working groups (WG). Overall, 40 experts discussed and reached a consensus on different relevant aspects, such as conditioning factors for initial funding and access, evaluation and access to RD-targeted therapies, funding of these therapies, and implementation of a new funding and access model. From these meetings, 50 proposals were defined and classified by their level of relevance according to the experts. A descriptive analysis of responses was performed for each proposal. Thereafter, experts completed another questionnaire where they ranked the 25 most relevant proposals according to their level of feasibility of being implemented in the SNHS. The most relevant and feasible proposals were to improve: process of referral of patients with RDs, control over monitoring mechanisms, and communication between healthcare professionals and patients.

**Conclusions:**

The FINEERR project may provide a starting point for stakeholders involved in the process of funding and access to RD-targeted therapies in Spain to provide the necessary resources and implement measures to improve both the quality of life and life expectancy of patients with RDs.

## Background

In recent decades, there have been substantial advances in the field of rare diseases (RDs), with an increase in social awareness, specific legislation approval, an increase in scientific activity, the exponential development of new therapies, and the creation of a community to give patients a voice and reduce their isolation[1, 2].

The development of European regulations served as a basis for the Member States to carry out specific measures to promote orphan medicinal products (OMPs) for these diseases [3]. This regulation has had a positive impact, reducing the average access time by 9 months and increasing the number of OMPs available. It is estimated that between 6 and 18% of the OMPs authorised since the implementation of the regulation until 2017 could have been the direct result of it [4]. Accordingly, some countries offer greater flexibility during the evaluation of these drugs (i.e., Germany) [5], while others use methods specifically designed to evaluate RD-targeted drugs (i.e., Australia, Scotland and England) [6–8]. In Spain, the number of authorized OMPs follows a similar trend to that of the European Medicines Agency [9]. However, only 44% of the medicines authorised in Europe until 2020 have been effectively marketed in Spain [10].

However, challenges regarding funding and access to RD-targeted drugs remain [11, 12]. There are currently between 5000 and 8000 diagnosed RDs, with only 700 having a specific drug [13, 14]. Furthermore, a marketing authorization does not necessarily mean that the drug is available or affordable to all Member States and in an equitable manner.

In Spain, slightly more than half of the OMPs authorized by the European Medicines Agency (EMA) are available to patients with public reimbursement, which can take up to 2 years [15, 16]. In Spain, price & reimbursement are taken on national level, however regions have increasing influence on the central price decision through their participation in Pricing Commission [17, 18]. This is, on average, 3–4 months longer than in neighbouring countries such as France and Italy, respectively [15, 16]. Moreover, the regulatory evidence supporting orphan drug’s authorization shows substantial uncertainties [19, 20].

In this context, the FINEERR project (Spanish acronym for Rare Disease Funding) aimed to carry out a multidisciplinary strategic discussion on the challenge posed by funding and access to RD-therapies in Spain and establish some proposals for improvement in this area. More specifically, this project aimed to gather information from 40 experts to reach a consensus on specific proposals to attain mid-term improvements on the matter and guide decision-making regarding optimal resource allocation for RDs-targeted therapies in the Spanish National Healthcare System (SNHS).

## Methods

The FINEERR project was organised around a CORE Advisory Committee (Table 1), which provided an overview, agreeing on the design and scope of the project, and help with the selection of members within each of four working groups (WG): WG1 discussed conditioning factors for initial funding and access, WG2 evaluation and access to RD-targeted therapies, WG3 funding of these therapies, and WG4 implementation of a new funding and access model (Table 2). WGs discussed and agreed upon relevant aspects of its specific theme to subsequently make proposals for improvement.

**Table 1:** Members of the CORE Advisory Committee

Name	Position
*CORE Advisory Committee*	
Alba Ancochea Díaz	Outgoing Director of the Spanish Federation of Rare Diseases (*Federación Española de Enfermedades Raras—FEDER*). Member of the International Rare Disease Research Consortium
Piedad Ferré de la Peña	Technical Advisor at the Ministry of Health
Olga Delgado Sánchez	President of the Spanish Society of Hospital Pharmacy (*Sociedad Española de Farmacia Hospitalaria—SEFH*). Head of the Pharmacy Service of Son Espases University Hospital, Palma de Mallorca
Nieves Martín Sobrino	Technical director of Pharmacy of the Castilla y León Health Management Department
Marta Trapero Bertran	Professor and researcher at the Catalonia International University (*Universitat Internacional de Catalunya – UIC*). Barcelona
Ignacio Málaga Diéguez	Outgoing President of the Spanish Society of Pediatric Neurology (*Sociedad Española de Neurología Pediátrica—SENEP*). Head of the Neuropediatrics Unit at the Asturias Central University Hospital
César Hernández García	Head of the department of medicines for human use of the Spanish Agency of Medicines and Medical Devices (*Agencia Española de Medicamentos y Productos Sanitarios—AEMPS*)

**Table 2:** Members of the working groups that participated in the FINEERR Project

WG-1	WG-2	WG-3	WG-4
**Aitor Aparicio**. Managing Director of the State Reference Center for Rare Diseases (Centro de Referencia Estatal de Enfermedades Raras—CREER) under the IMSERSO. Burgos **Antonia Campolongo**. Nurse, Movement Disorders Unit, Neurology Service, Sant Pau Hospital. Barcelona **Emili Esteve**. Director of the Technical Department of Farmaindustria **Atanasio García**. General Director of Benefits and Pharmacy. Department of Health and Consumption. Government of the Balearic Islands **Pablo Lapunzina**. Head of the INGEMM-IdiPAZ Research Group. La Paz University Hospital. Madrid **Álvaro Lavandeira**. President of the Institute for Health Research and Training. Advisor in Pharmaceutical Law at the Madrid Bar Association (Ilustre Colegio de Abogados de Madrid—ICAM) **Alfonso Macaya**. Head of Pediatric Neurology at Vall d'Hebron University Hospital. Barcelona **José María Millán**. Researcher at the Health Research Institute La Fe (Instituto de Investigación Sanitaria—IIS La Fe). Valencia **Manuel Posada**^a^. Director of the Research Institute of Rare Diseases of the Carlos III Health Institute (Instituto de Salud Carlos III—ISCIII). OPIS Research Professor in Rare Diseases **Ignacio Málaga Diéguez**. Outgoing President of the Spanish Society of Pediatric Neurology (Sociedad Española de Neurología Pediátrica—SENEP). Head of the Neuropediatrics Unit at the Asturias Central University Hospital. Member of the CORE Advisory Committee of the FINEERR project	**Reyes Abad**. Head of Pharmacy of the Miguel Servet University Hospital. Zaragoza **Jorge Camarero**. Vice President of PAREXEL Consulting. Former scientific evaluator of the Spanish Agency of Medicines and Medical Devices (Agencia Española de Medicamentos y Productos Sanitarios—AEMPS) **Francisco Dolz**. Manager of Dr Peset University Hospital. Valencia **Víctor Jiménez**. Head ofthe Hematology and Hemotherapy Service, La Paz University Hospital. Madrid **Mencía de Lemus**. President of the Spinal Muscular Atrophy Foundation (Fundación Atrofia Muscular Espinal- FundAME) **Isabel Martín**. Head of the Pharmacy Service of the University Hospital Complex. A Coruña **Jorge Matías-Guiu**. Director of the Institute of Neurosciences. Head of the Neurology Service of the San Carlos Clinical Hospital. Madrid **Jorge Mestre**. Independent economic consultant. Associate Professor at Carlos III University. Madrid **Irene Beatriz Zschaeck**. Neuropsychologist at Sant Joan de Déu Hospital. Barcelona **Marta Trapero Bertran**. Professor and researcher at the Catalonia International University (Universitat Internacional de Catalunya – UIC). Barcelona. Member of the CORE Advisory Committee of the FINEERR project	**José Javier Castrodeza Sanz**. Professor of Preventive Medicine and Public Health at the University of Valladolid **Carlos García Collado**. Deputy Director General of Pharmacy and Services in the Andalusian Health Service **Pedro Gómez Pajuelo**. Former Secretary General of the ONT and former Deputy Director General of Pharmacy **Caridad Pontes García**. Medicines Manager of the Catalan Health Service Santiago de la Riva. Vice-president of the Spanish Federation of Rare Diseases (Federación Española de Enfermedades Raras—FEDER) **Pedro Luis Sánchez García**. Director of the Research Department of Farmaindustria **Luis Verde Remeseiro**. Manager of the Integrated Health Area of A Coruña SERGAS. Academic Director of the Master’s Degree in Healthcare and Hospital Management at IFFE Business School **Olga Delgado Sánchez**. President of the Spanish Society of Hospital Pharmacy (Sociedad Española de Farmacia Hospitalaria—SEFH). Head of the Pharmacy Service of Son Espases University Hospital. Palma de Mallorca. Member of the CORE Advisory Committee of the FINEERR project	**Soledad Cabezón Ruiz**. Former Member of the European Parliment on behalf of the Spanish Socialist Workers’ Party (Partido Socialista Obrero Español – PSOE). Cardiologist **José Martínez Olmos**. Professor at the Andalusian School of Public Health (Escuela Andaluza de Salud Pública—EASP) **Antoni Montserrat Moliner**. Member of the Board of Directors of ALAN—MALADIES RARES LUXEMBOURG **Rubén Moreno Palanques**. Senator on behalf of the Popular Party (Partido Popular—PP). Member of the Health and Consumption Commission **Ana Pastor Julián**. Member of the Spanish Parliament on behalf of the Popular Party (Partido Popular—PP). Member of the Health and Consumption Commission. Second Vice-President of the Congress of Deputies **Ana Prieto Nieto**. Member of the Spanish Parliament on behalf of the Spanish Socialist Workers' Party (Partido Socialista Obrero Español—PSOE). Spokesperson in the Health and Consumption Commission. First Secretary of the Interior Commission **Julio Sánchez Fierro**. Lawyer and Health Sciences PhD **Juan Luis Steegmann**. Member of the Spanish Parliament on behalf of VOX. Spokesperson in the Health and Consumption Commission **Alba Ancochea Díaz**. Outgoing Director of the Spanish Federation of Rare Diseases (Federación Española de Enfermedades Raras—FEDER). Member of the International Rare Disease Research Consortium. Member of the CORE Advisory Committee of the FINEERR project Ignacio Málaga Diéguez. Outgoing President of the Spanish Society of Pediatric Neurology (Sociedad Española de Neurología Pediátrica—SENEP). Head of the Neuropediatrics Unit at the Asturias Central University Hospital. Member of the CORE Advisory Committee of the FINEERR project

^a^MP participated in this WG on a personal account and as a professor in Rare Disease Epidemiology and Research and was therefore not representing or acting on behalf of the Carlos III Research Institute

The members of the WGs were multidisciplinary, with different profiles chosen to represent all the stakeholders involved in the process of funding and access, such us clinicians with different specialties, pharmacists, researchers, health law specialists, health economists, health managers, administrators, and politicians. Moreover, all the WGs, as well as the CORE Advisory Committee, included the participation of a patient representative. Experts were selected based on their experience in the field of RDs and for obtaining a representative geographical sample (9 Autonomous Communities).

The project was led by Weber (Health Economics Research and Consulting Centre), which was responsible for the literature review, the organization and coordination of each expert group, the development and analysis of diagnostic questionnaires, and the development of the final project report. The members of the Weber Foundation who participated in the project were health economists.

The members of each WG were convened for an online session held between September 2020 and January 2021. Before each session, the experts in WG1-3 completed an online questionnaire regarding the situation of RD-targeted therapies in Spain. WG4, with a more political profile, had a different methodological approach, since they focused on debating and reflecting on the implementation of a new financing and access model for therapies aimed at RDs in Spain, based on the recommendations made throughout the project up to that point. For this reason, the members of WG4 did not carry out an explicit diagnosis questionnaire of the current situation.

Experts received a specific pre-reading material based on a literature review, which included the regulatory framework, clinical conditioning factors, research, evaluation and funding, follow-up mechanisms, among others. Both the questionnaire and the pre-reading material were previously validated by the CORE Advisory Committee. Moreover, at least one member of the CORE Advisory Committee participated in each WG, providing the project overview in the particular discussion.

The four online sessions were designed as spaces for multidisciplinary discussion. Each session was organized in small debate groups (3–4 people) to discuss relevant aspects of RD-targeted therapies in Spain, with the aim of sharing thoughts and proposing recommendations for improvement in each area. Sessions were concluded by sharing recommendations with all FINEERR experts to further refine, complete, and validate them.

A list of 50 recommendations for action, grouped into 9 specific areas, was obtained from the 4 online sessions. These were ordered according to the mean and standard deviation of the scores obtained from an online questionnaire, where experts of the CORE Advisory Committee and WG1-3 were asked to rate each recommendation on a scale of 0 (not relevant at all) to 10 (very relevant) and provide a reason for such score. Finally, to identify the most relevant and feasible recommendations for action, all experts ranked the 25 most relevant identified recommendations using another questionnaire where they were asked to classify each recommendation as having high or low feasibility of implementation in the SNHS and to consider the barriers to their implementation. Weber performed a descriptive analysis of this second questionnaire, translating the results into diagrams by thematic area.

## Results

### Aspects relevant to the funding and access to RD-targeted therapies: literature review

#### General regulatory framework: incentives at a European level

In 2000, the European Commission approved a specific regulation for the promotion of RD-targeted therapies through the enactment of Regulation No. 141/2000 [21] and Regulation No. 1901/2006 [4]. The European Union (EU) currently defines an orphan drug as a therapy that meets the following criteria [13]: I) intended for the diagnosis, prevention or treatment of a life-threatening or chronically debilitating condition affecting not more than five in 10 thousand persons in the Community when the application is made, or intended for a condition that without incentives it is unlikely that the marketing of the medicinal product would generate sufficient return to justify the necessary investment; and II) there exists no satisfactory method of diagnosis, prevention or treatment of the condition in question that has been authorised in the EU or, if such method exists, the medicinal product will be of significant benefit to those affected by that condition.

A drug meeting these requirements would benefit from a series of advantages, including scientific advice at a reduced cost, additional administrative assistance for small and medium-sized enterprises (SMEs), and additional R + D funding, among others [22].

#### Clinical conditioning factors

Diagnosis is the first barrier faced by patients suffering from a RD, the main cause being a lack of knowledge, and having a physical, psychological, and emotional impact [23]. In Spain, it has been estimated that 7.6% of patients with a RD have a non-definitive diagnosis and that 3.2% have not been diagnosed [23]. These problems with diagnosis may stem from lack of knowledge about rare diseases, lack of academic education and lack of information pointed out by both primary care physicians and specialists [24]. An accurate diagnosis benefits patients by improving prognosis, reducing isolation, or enhancing socio-sanitary care, among others [25].

Another important aspect of access to RD-targeted therapies is the correct management of patient care, which requires a care network that covers all the necessary social and healthcare processes. Accordingly, Spain has a National Strategy on RDs and several regional strategic plans which aim to improve the prevention, diagnosis, and care of people with RDs, as well as other initiatives that directly affect RDs [26–29]. Likewise, the SNHS centers, services, and reference units (CSUR) are key in the care of people with RDs [30]. There are currently 279 CSURs in Spain associated with 70 different diseases (many of them RDs), in 13 out of 17 Autonomous Communities.

#### Criteria for funding RD-targeted therapies

OMPs are centrally authorized by the EMA. Thereafter, public funding in each country is decided by the member states, based on different criteria and procedures. In Spain, RD-targeted therapies follow the same funding and access procedure as other drugs do. These are clinically and economically evaluated through therapeutic positioning reports (TPR), whose governance process has been recently improved [31].

When it comes to making a decision on funding RD-targeted therapies, three types of criteria are usually considered: clinical criteria, including the absence of alternative therapies, the severity of the disease, and the possibility of changing the course of the disease; economic criteria including the budget impact, opportunity costs, or the sustainability of the healthcare system; and humanistic criteria, which are based on concepts of equity, justice, and the Rule of Rescue or moral imperative to save a life that is in imminent danger, as a type of solidarity [32].

In Spain, the law establishes that funding drugs through the SNHS is possible on account of criteria such as: the severity, duration, and impact of the disease; individual group needs; the therapeutic and social value of the drug and its clinical benefit; the rationalization of public spending and budget impact; the existence of therapeutic alternatives; and the degree of innovation of the drug. Some of these criteria would particularly affect patients suffering from RD [33].

#### The role of patients and patient associations

Including patients and patient associations may generate a positive impact, improving knowledge and risk perception accuracy for each disease, facilitating the selection of options in accordance with patient values, and minimizing conflicting sensations generated during the process [34]. In this respect, projects actively involving patients in the decision-making process have proliferated in recent years.

For example, three representatives of RD patient associations were recently included in the OMP Committee of the EMA, which is responsible for the designation of orphan drugs, advising on the development and implementation of an orphan drug policy in the EU, and the development of detailed guidelines related to these drugs [4].

Meanwhile, different RD patient associations seek to improve the quality of life of patients by creating networks to share experiences, improving research and knowledge on RDs, and being key in the development of policies and strategies at a European level (e.g., Rare Diseases Europe—EURODIS) or at a national level (e.g., Spanish Federation on Rare Diseases—*FEDER*) [35, 36].

### Diagnostic on the current situation

**Fig. 1:**
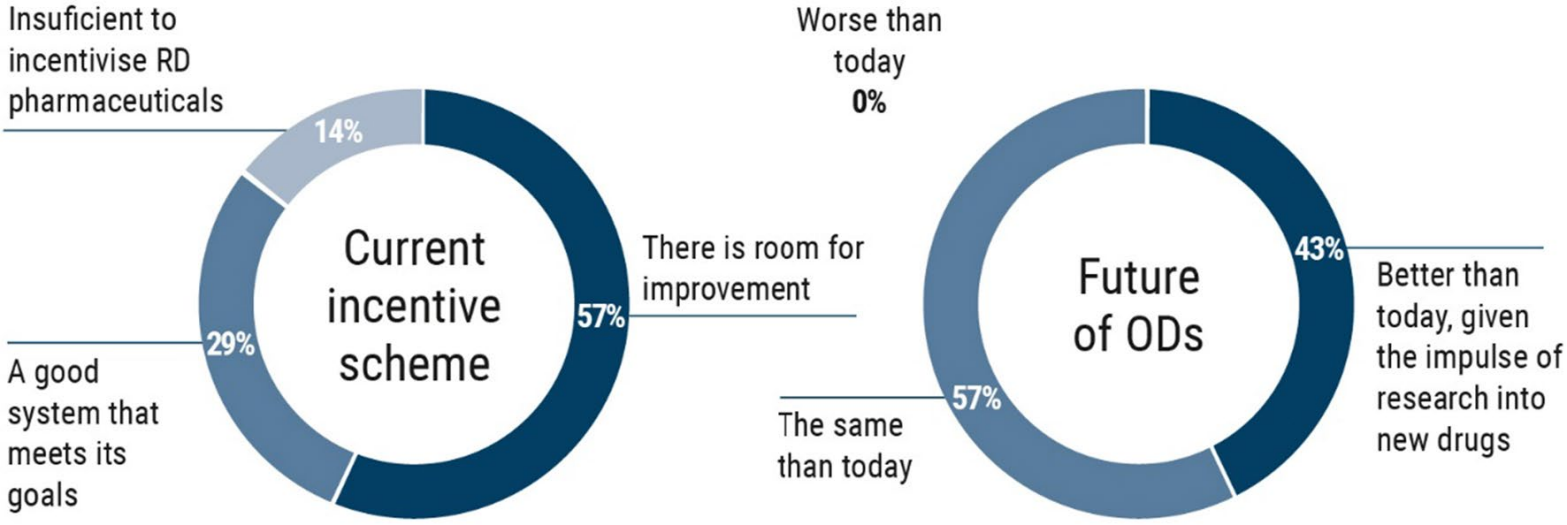
Diagnosis of the current incentives system and the future of OMPs in Spain (n = 7)

According to the results of the questionnaires, 57% of the experts in the CORE Advisory Committee believe there is room for improvement in the current system of incentives for the development of RD-targeted therapies, while 29% believe the system is appropriate, and 14% believe it is insufficient. Looking into the future, 57% believe that the current situation will not change, and 43% believe it will improve given advances in R + D regarding new drugs. None of the experts believes that the situation will get worse (Fig. 1).

**Fig. 2:**
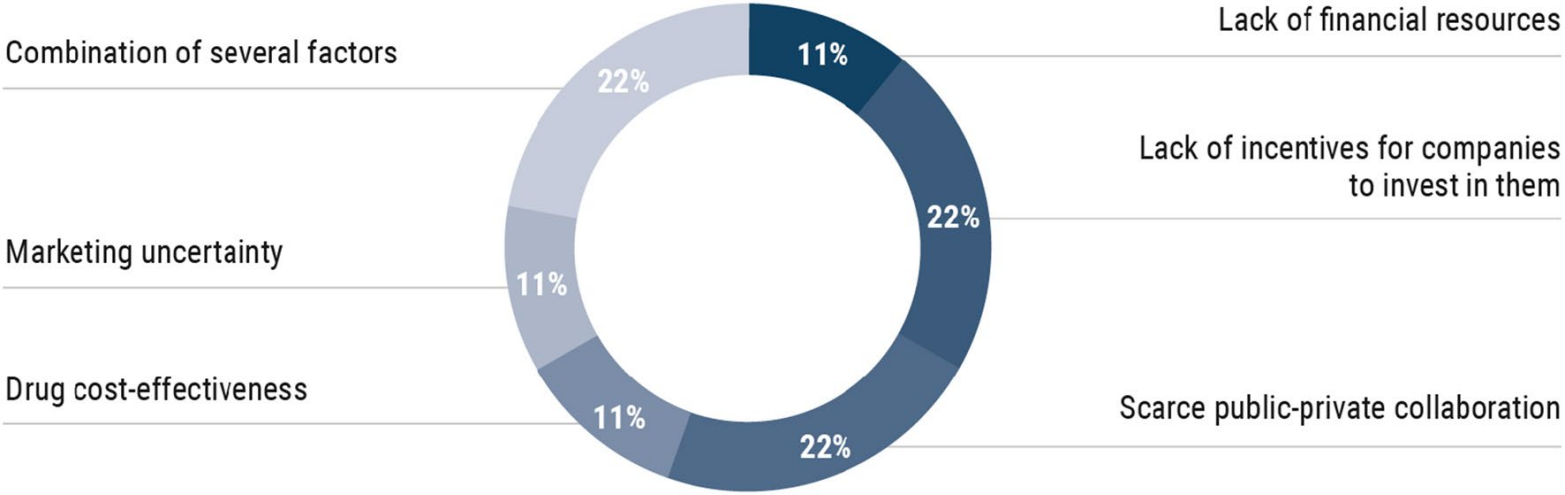
Main problems for the development of new RD drugs (n = 9)

Regarding the development of new RD therapies, experts consider up to 6 different obstacles: lack of incentives for companies to invest (22%), poor public–private collaboration (22%), a combination of several aspects that range from trial design to incentives demanded by companies that are non-sustainable for the healthcare system or lack of knowledge about the disease (22%), lack of financial resources (11%), lack of drug profitability (11%) and uncertainty in marketing conditions (11%) (Fig. 2).

**Fig. 3:**
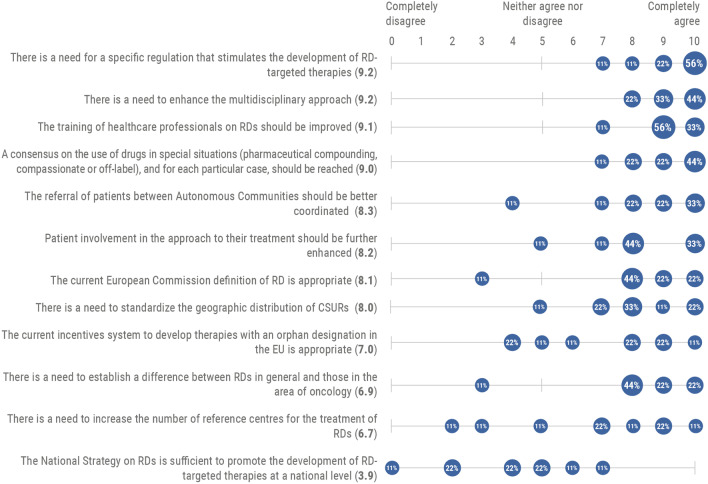
Diagnosis of the legislation and approach to RDs in Spain (n = 9). Rate each item on a 0–10 scale

When the experts were asked about their degree of agreement with several issues regarding the regulation of RD therapies and their approach in Spain, most experts agreed there is a need for a specific regulation to stimulate their development. Meanwhile, there is also a need to enhance the multidisciplinary approach, improve the training of healthcare professionals, and reach a consensus on the use of RD therapies in special situations and for each particular case. However, no clear consensus was reached on increasing the number of reference centers nor on the National Strategy on RDs being sufficient to promote the development of these therapies (Fig. 3).

Regarding the economic evaluation of RD-targeted therapies from a social perspective, most experts agreed the patient should be more involved in the evaluation process (mean: 8.2). Moreover, some of them believed the willingness-to-pay threshold should be higher for RD-targeted therapies than for those of more prevalent diseases (7.8), but there was no firm agreement on that. Some experts questioned the validity of current economic evaluation models evaluating RD-targeted therapies in Spain (5.0).

The experts believed the main challenge for funding RD-targeted therapies is the uncertainty in terms of outcomes and the number of patients (43%). However, other obstacles such as the lack of specific criteria for decision-making (29%) or the availability of financial resources (14%) are also considered a priority.

Regarding the current pricing and funding times for RD-targeted therapies in Spain, most experts believe they are too long, and important bottlenecks should be addressed (47%), while others believe efforts should be made to reduce them (41%), yet 12% consider them reasonable, given the circumstances.

### Analysis of proposals

#### Relevance of proposals

The 50 proposals for improvement that emerged from the WG discussions were grouped into 9 areas: research (7 proposals), socio-sanitary care (6), access improvement (10), evaluation (4), transparency (4), patients (4), price and funding (4), funding methods (5), and monitoring mechanisms (6).

**Fig. 4:**
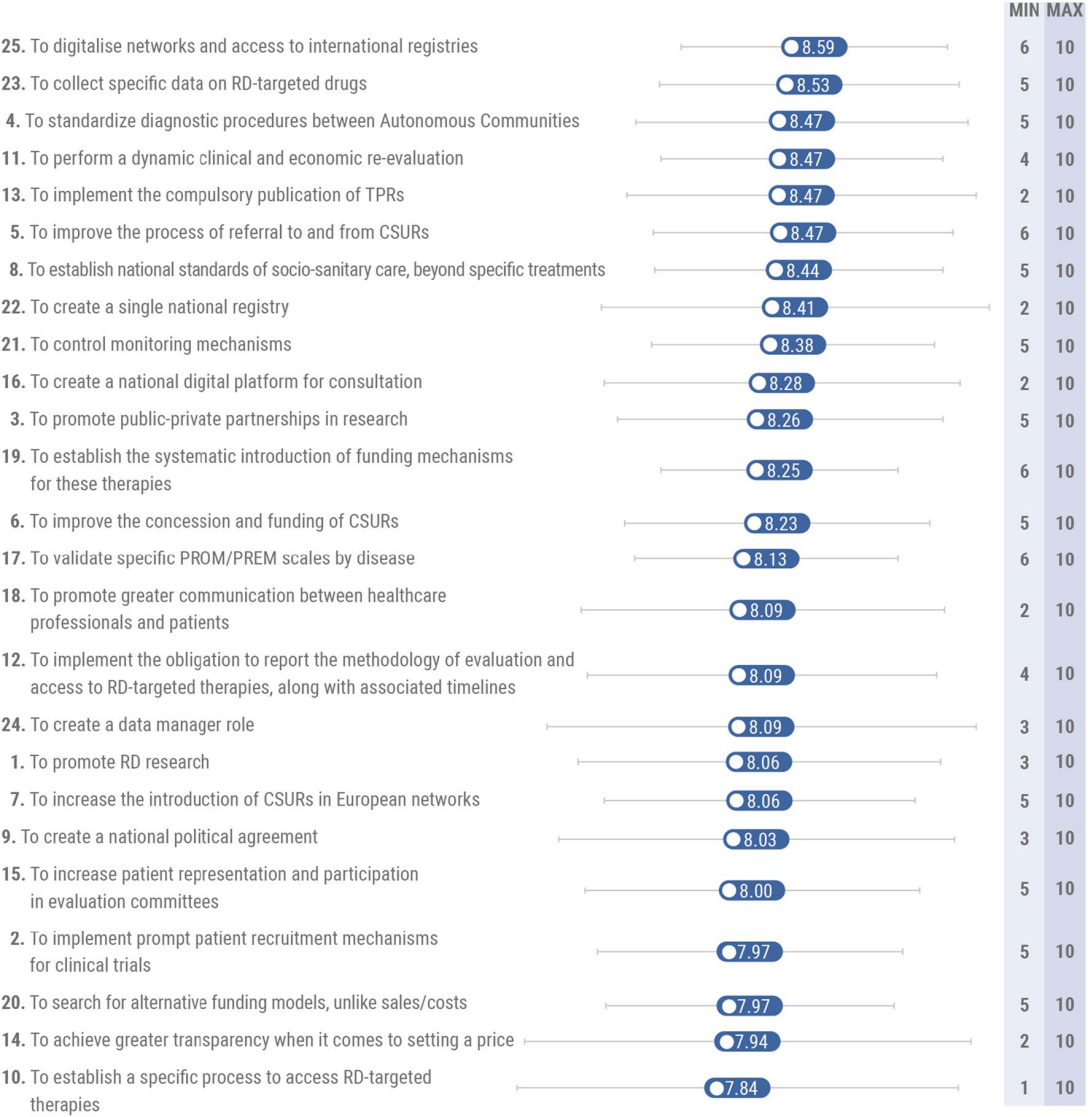
The 25 most relevant proposals for action as rated by experts

Overall, the most relevant proposal was to digitalize networks and access to international registries, obtaining a score of 8.59 out of 10, followed by the proposal to collect specific data on OMPs (8.53) and standardize diagnostic procedures between Autonomous Communities (8.47) (Fig. 4).

Conversely, of the 25 most relevant proposals, the three with the lowest score were to search for alternative funding models, achieve greater transparency when it comes to setting a price, and establish a specific process to access RD-targeted therapies, obtaining an average score of 7.97, 7.94, and 7.84, respectively (Fig. 4). It is noteworthy that 4 of the 10 most relevant measures were under the monitoring mechanisms area (i.e., to digitalize networks, collect specific data on OMPs, create a single national registry, and control monitoring mechanisms). Moreover, 3 measures in the top 10 were under the socio-sanitary care area (i.e., to standardize diagnostic procedures between Autonomous Communities, improve the process of referral to and from CSURs, and establish national standards of socio-sanitary care). A full definition of the 25 most relevant recommendations has been provided in the “Appendix 1” section.

Regarding the level of consensus among experts, of the 25 most relevant proposals, those with the greatest consensus (lower standard deviation) were to establish the systematic introduction of funding mechanisms (SD: 1.11), validate specific PROMs/PREMs scales by disease (SD: 1.24), and digitalize networks (SD: 1.27). Conversely, those with the lowest consensus were to achieve greater transparency when it comes to setting a price (SD: 2.11), establish a specific process to access RD-targeted therapies (SD: 2.08), and create a data manager role (SD: 2.04) (Fig. 4).

**Fig. 5:**
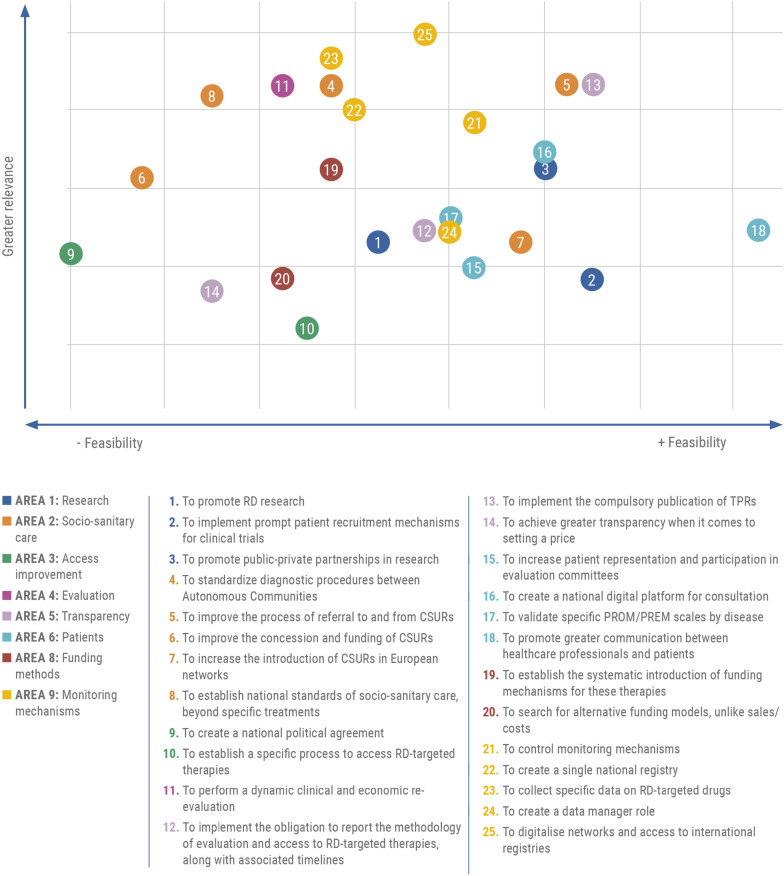
The 25 proposals according to relevance and feasibility level, by areas

Moreover, the areas with the greatest number of proposals in the top 25 were those associated with socio-sanitary care and monitoring mechanisms, with five proposals for improvement each (Fig. 5).

#### Feasibility of implementing proposals

Regarding the feasibility of implementing the 25 most relevant proposals as rated by the FINEERR experts, the most feasible was to promote greater communication between healthcare professionals and patients, given that 97.5% considered this measure highly feasible. Other highly feasible proposals were to implement the compulsory publication of TPRs (among the 10 most relevant proposals) and implement prompt patient recruitment mechanisms for clinical trials.

In contrast, only 25% of the experts considered the proposal to create a national political agreement feasible, given the political situation in Spain. In this regard, some experts believe that access decisions should not be political, but technical, and based on guarantees of quality, efficacy, safety, and sustainability, under charity, distributive justice, and equity criteria. Moreover, the proposal to improve the concession and funding of CSURs was considered highly feasible by only 32.5% of experts, given the decentralization of competencies between Autonomous Communities in Spain, and to establish national standards of socio-sanitary care by only 40%, given that resources exceed the capacity of the healthcare system itself.

Experts with political profile (WG4) also considered that the most feasible measure was the improvement in the professional-patient communication, followed by data monitoring (single registry, national platform for consultation, data manager and specific collection for RDs). On the contrary, the least feasible measured would be the application of alternative financing models not linked to sales or cost, where they largely disagree with the rest of WGs. Half of the experts of WG4 believe that it is relatively feasible to reach a political agreement at the national level.

The analysis by areas showed that all proposals in the patient area obtained the highest mean feasibility score, while their mean relevance score was moderate. Moreover, the research and transparency areas also included highly feasible proposals. Conversely, the least feasible proposals were within the access improvement and socio-sanitary care areas (Fig. 5).

The analysis of the 25 most relevant proposals by relevance and feasibility combined showed that the most relevant and feasible proposals were to implement the compulsory publication of TPRs to guarantee transparency (relevance: 8.47; feasibility: 80.0%)^1^; improve the process of referral to and from CSURs to enhance the quality and equity if the healthcare system (relevance: 8.47; feasibility: 77.5%); control monitoring mechanisms, determining real-world data that should be collected and their use (relevance: 8.38; feasibility: 67.5%); and promote greater communication between healthcare professionals and patients to increase understanding on the matter (relevance: 8.09; feasibility: 97.5%).

## Discussion

The FINEERR project was conceived as a space for strategic debate on the currently relevant challenge of funding and access to RD-targeted therapies in Spain. The multidisciplinary nature of the WGs provided a multidimensional approach to the matter, integrating micro-, meso-, and macro-level views from 40 different experts. These experts contributed with their experience, vision, and training, to the analysis of the problem, discussion of obstacles and triggers for change, and specific proposals to improve funding and access to RD-targeted therapies in the SNHS.

The proposals that stand out for their relevance are those focused on improving the digitalization of the system and the dynamic re-evaluation of medicines, the standardization of processes to achieve greater territorial equity (i.e., diagnosis, referral, healthcare, socio-sanitary care, and treatment access), the transparency of decision-making processes, enabling funding and access to OMPs, and making TPR methods and timings public.

This is, however, a very complex issue with multiple factors and stakeholders involved at different levels. Most proposals require political will and the elimination of reservations and barriers to implement them. They also need efforts by the pharmaceutical industry in terms of transparency. Moreover, general changes in the system would benefit RD therapies in most cases, yet sometimes specific actions are necessary. A sign of optimism for change is that the experts conforming WG4, with a political profile, consider it relatively feasible to reach a political agreement at the national level to improve access to OMPs.

Other studies have also collected a series of proposals in the field of RDs, such as the one published by the Expert Group on Rare Diseases of the European Commission in 2016. This document urged member countries to take measures which have also been proposed in the present study such as to promote the exchange of information between patients and healthcare professionals or include specialized centers in European Reference Networks on Rare Diseases [37]. Another noteworthy document, signed by more than 70 organizations, scientific societies, patient associations, foundations, and pharmaceutical industries in 2017, included 11 proposals for improvement in the field of RDs in Spain. Some of these proposals were also obtained in the FINEERR project, such as to implement a comprehensive care model, standardize diagnosis between Autonomous Communities, or promote research on RD-targeted therapies [38].

Nevertheless, the FINEERR project takes a more technically detailed approach and broadens the scope of the analysis, including economic aspects, and proposals for improvement that include not only management-based proposals or clinical proposals aiming to improve diagnosis or assistance, but also proposals aiming to improve funding and access to RD-targeted therapies (i.e., to perform a dynamic clinical and economic re-evaluation, collect specific data on OMPs, or digitalize networks and access to international registries).

The time is right for change, as 20 years have passed since the specific regulation to promote OMPs was implemented in Europe and the current transition towards reformulating incentives at an EU level [39]. Some of the problems identified in our study on the development of new drugs for rare diseases, such as lack of incentives for companies to invest on them or the scarce public–private collaboration, could serve as a starting point for European policy makers on a reform of access to orphan drugs. In addition to this, there are other trends of change towards greater precision and costs of treatment and a greater digitalization of the healthcare system [40, 41]. Taken together, and in the context of the biggest economic, social, and healthcare crisis of the last century, largely due to the COVID-19 pandemic, a change is deemed necessary. Accordingly, we must identify bottlenecks and learn from good practices at national and international levels to optimize the healthcare system in terms of efficiency, equity, and sustainability.

Several limitations should be considered when interpreting the results of this study. First, results are based on the opinions of a group of participants, which can be biased by their own experience and human nature itself. Nevertheless, participants were up to date on the state of funding and access to RD-targeted therapies in Spain and had a broad professional background. Second, a convenience sample of experts was selected for this study, and the small sample size (n = 40) may not be representative of the overall problem. Furthermore, the experts were given the option to provide a justification for each of the questionnaire scores. In addition, this project has mainly focused on the prevention, diagnosis and treatment of rare diseases, without considering other potential relevant strategies such as surgery, radiation, diet, devices, etc. Finally, given the time period of the study (the year 2020–2021) the latest regulatory and administrative developments in Spain were not accounted for (i.e., the compulsory publication of TPRs has already been implemented [31]).

## Conclusion

The FINEERR project may provide a starting point for stakeholders involved in the process of funding and access to RD-targeted therapies to provide the necessary resources and implement measures to improve both the quality of life and life expectancy of patients with RDs.

A coordinated effort is required from the different stakeholders, including the pharmaceutical industry, with clear leadership of healthcare authorities, to allow the overall healthcare system to meet the technical, political, economic, and social challenges ahead. Future studies should explore this issue further to assess how best to implement these recommendations over time.

## Data Availability

The data used in this study have been generated by the expert committees of the project. We can share them, in Excel format and Spanish language, upon request.

## Appendix 1: Full definitions of the 25 most relevant recommendations

1. *To promote RD research.* This proposal aims to improve the structure of the framework of research incentives offered by the member states. Research on the development of new molecules, compounds, and advanced therapies (with drug repositioning) should be promoted to a greater extent. Moreover, research centers should be encouraged to carry out research on RD-targeted therapies, allocating more funds to improve and motivate RD research.
2. *To implement prompt patient recruitment mechanisms for clinical trials.* Good practices used in several hospitals should be implemented for the prompt recruitment of patients in clinical trials at a national level. This would place Spain as an important RD research center in Europe.
3. *To promote public–private partnerships in research.* Co-development models should be promoted largely, with the Administration participating from the early stages in the design of clinical trials (similar to the current development of vaccines against COVID-19).
4. *To standardize diagnostic procedures between Autonomous Communities.* This proposal aims to design and coordinate procedures of patient referral between centers at a national level. Moreover, it aims to implement a uniform screening system between Autonomous Communities, neonatal-based at first and population-based in the foreseeable future. This screening would be selective, focusing on diseases with evidence that prompt treatment associated with early detection can change the course of the disease. Moreover, the standardization of the screening procedure should be at a national and European level. A more coherent framework for neonatal screening and detection is needed to allow a prompt diagnosis.
5. *To improve the process of referral to and from CSURs.* Administrative procedures that delay referrals should be reduced. Moreover, more information should be provided for patients and healthcare professionals such that referral times are minimized.
6. *To improve the concession and funding of CSURs.* This proposal aims to determine an appropriate CSUR policy that is not based on defining evaluation criteria. A plan for the distribution and definition of the needs that should be met by CSURs would be desirable, including the designation of new centers on a competitive basis and not so much at the proposal of the Autonomous Communities. Moreover, a territorial equity factor should also be included. This would apply to both new CSURs and new CSUR categories.
7. *To increase the introduction of CSURs in European networks.* This proposal aims to introduce more CSURs in European Reference Networks to take advantage of the knowledge generated and improve treatments in the field of RDs, where the variability of a small number of cases hampers healthcare assistance.
8. *To establish national standards of socio-sanitary care, beyond specific treatments.* This proposal aims to set standards of care at a national level regarding caregiving, rehabilitation, psychological assistance, and other socio-sanitary care needs of patients with RDs, beyond the pharmaceutical treatment.
9. *To create a national political agreement.* Given the relevance of proposals and to improve access in the field of RDs, a national agreement between all political parties would be desirable, beginning with those proposals achieving a greater consensus. Moreover, an agreement between stakeholders is also necessary to improve patient-access to therapies.
10. *To establish a specific process to access RD-targeted therapies.* The aim of this proposal was to establish a specific procedure for the prompt access to RD-targeted therapies that would guarantee a fast-track for potentially very innovative therapies and diseases with important unmet needs (i.e., not necessarily for all OMPs). This fast-track should make repositioning and evaluation of better therapeutic options possible. Moreover, representatives of all stakeholders involved should reach a consensus on the matter.
11. *To perform a dynamic clinical and economic re-evaluation.* This proposal aims to promote the dynamic re-evaluation of innovation based on new information, which is especially relevant when uncertainty is high. Data collection should be designed according to the type of uncertainty (clinical or economic) as well as the most appropriate outcome-based funding model. To do so, adequate data-collection procedures on benefits, safety, direct and indirect costs, and budget impact should be promoted to treat the greatest number of diseases and patients possible.
12. *To implement the obligation to report the methodology of evaluation and access to RD-targeted therapies, along with associated timelines.* This proposal aims to improve the transparency of pharmacotherapeutic commissions regarding evaluation times and procedures, at hospital and Autonomous Community levels, to improve the access to OMPs at healthcare centers.
13. *To implement the compulsory publication of TPRs.* The publication of TPR should be compulsory from the moment it is carried out.
14. *To achieve greater transparency when it comes to setting a price.* This proposal aims to improve transparency in setting drug prices in terms of how the price is set and the contribution of public research to the development of new drugs. This transparency should be provided both by the pharmaceutical industry (i.e., disclosing the costs of drug research and the manufacturing process), and by the administrations (i.e., disclosing price-setting factors).
15. *To increase patient representation and participation in evaluation committees.* The representation of patients in drug evaluation committees include patients affected by the disease and patients as a community, providing a more neutral view from a societal perspective. Moreover, this proposal aims to promote and involve patients within these committees, as they are especially aware of the impact of RDs on quality of life. To this end, patient education is very important. The evaluation should be carried out by independent experts, without conflicts of interest beyond the fact that the opinion of patients should be taken into account.
16. *To create a national digital platform for consultation.* This proposal aims to improve the information that patients receive in the field of RD. To do so, the creation of a national digital platform with information on RDs and available CSURs is suggested.
17. *To validate specific PROM/PREM scales by disease.* This proposal aims to design and validate RD-specific PROMs/PREMs scales in our country, with relevant and direct inputs from patients.
18. *To promote greater communication between healthcare professionals and patients.* This proposal aims to improve the communication between RD experts and patients in both directions. To do so, the promotion of open days or monographic days on RDs is suggested.
19. *To establish the systematic introduction of funding mechanisms for these therapies.* This proposal aims to automatically link every RD-targeted therapy to a funding mechanism (e.g., risk-sharing agreements, agreements based on health outcomes, expenditure ceilings, etc.). A requirement satisfaction guide could be established to screen therapies for the use of one funding mechanism or another. Moreover, this screening mechanism would use inputs such as outcome uncertainty and lack of responder identification, among others.
20. *To search for alternative funding models, unlike sales/costs.* Alternative funding mechanisms for RD-targeted therapies should be explored and implemented (e.g., financing mechanisms used for antibiotics against multidrug-resistant germs). The return on investment would be more closely linked to a series of payment models for reaching certain milestones, and a flat fee for having the therapy available and not for the sales/use of this therapy (availability subscription model). Hybrid systems could also be considered.
21. *To control monitoring mechanisms.* This proposal aims to advance the control model of monitoring mechanisms. This model should determine which real-world data should be collected and its use, considering the cost of opportunity in terms of resources and time. The roles, policies, and measures should be specified from the start to make an efficient use of monitoring mechanisms.
22. *To create a single national registry.* This proposal aims to advance in the implementation of an effective single national registry based on the one developed by the Carlos III Research Institute. This registry should be publicly funded and supported by regional registries providing standardized and well-defined healthcare data and criteria. The extended use of Valtermed^2^ platform is suggested.
23. *To collect specific data on RD-targeted drugs.* Data from routine clinical practice should be collected, as well as other information reported directly by patients. Information on direct and indirect costs and quality of life measures of patients and their caregivers should also be stored. In addition, information to aid research and re-evaluation of therapies should be collected. Data should be compatible with Big Data (structured information), involving statistical data analysis or knowledge engineering experts.
24. *To create a data manager role.* This proposal aims to standardize the data manager role in each healthcare center of the SNHS, to improve treatment and data administration, relieving physicians of administrative tasks.
25. *To digitalize networks and access to international registries.* The necessary connections must be established to be able to share information at a national and international level. According to the European pharmaceutical strategy, the interoperability between national and European registries is essential. The national registry should move beyond the regional and national level and be included in the European platform of RD registries.

## Abbreviations

CSUR: SNHS centres, services, and reference units
EMA: European Medicines Agency
EU: European Union
FINEERR: Spanish acronym for Rare Disease Funding
OPM: Orphan Medicinal Product
R + D: Research and development
RD: Rare disease
SME: Small and medium-sized enterprise
SNHS: Spanish National Healthcare System
TPR: Therapeutic position reports
WG: Working group

## Footnotes

1 It is worth noting that the TPR’s consolidation plan, approved in parallel of this FINEERR study, already considers the mandatory publication of these reports.
